# Cardioprotective Signaling Pathways in Obese Mice Submitted to Regular Exercise: Effect on Oxysterols

**DOI:** 10.3390/ijms231810840

**Published:** 2022-09-16

**Authors:** Caroline Barau, Shirin Leick, Claudio Caccia, Lolita Portal, Valerio Leoni, Philippe Le Corvoisier, Didier Morin, Bijan Ghaleh, Sandrine Pons

**Affiliations:** 1 Inserm U955-IMRB, UPEC, Ecole Nationale Vétérinaire d’Alfort, F-94010 Créteil, France; 2 Plateforme de Ressources Biologiques, APHP, Hôpitaux Universitaires Henri Mondor, F-94000 Créteil, France; 3 Fondazione IRCCS—Istituto Neurologico Carlo Besta, 20131 Milano, Italy; 4 Laboratory of Clinical Biochemistry, Hospital Pius XI of Desio, ASST-Brianza, University of Milano-Bicocca, 20126 Monza, Italy; 5 Department of Medicine and Surgery, University of Milano-Bicocca, 20126 Monza, Italy; 6 INSERM CIC 1430, APHP, Hôpitaux Universitaires Henri Mondor, F-94000 Créteil, France

**Keywords:** regular exercise, obesity, pro-survival kinases, protein phosphatases, oxysterols, mitochondrial permeability transition pore

## Abstract

Exercise induces cardioprotection against myocardial infarction, despite obesity, by restoring pro-survival pathways and increasing resistance of mitochondrial permeability transition pore (mPTP) opening at reperfusion. Among the mechanisms involved in the inactivation of these pathways, oxysterols appear interesting. Thus, we investigated the influence of regular exercise on the reperfusion injury salvage kinase (RISK) pathway, oxysterols, and mitochondria, in the absence of ischemia-reperfusion. We also studied 7β-hydroxycholesterol (7βOH) concentration (mass spectrometry) in human lean and obese subjects. Wild-type (WT) and obese (ob/ob) mice were assigned to sedentary conditions or regular treadmill exercise. Exercise significantly increased Akt phosphorylation, whereas 7βOH concentration was reduced. Moreover, exercise induced the translocation of PKCε from the cytosol to mitochondria. However, exercise did not affect the calcium concentration required to open mPTP in the mitochondria, neither in WT nor in ob/ob animals. Finally, human plasma 7βOH concentration was consistent with observations made in mice. In conclusion, regular exercise enhanced the RISK pathway by increasing kinase phosphorylation and PKCε translocation and decreasing 7βOH concentration. This activation needs the combination with stress conditions, i.e., ischemia-reperfusion, in order to inhibit mPTP opening at the onset of reperfusion. The human findings suggest 7βOH as a candidate marker for evaluating cardiovascular risk factors in obesity.

## 1. Introduction

Obesity is a serious risk factor for cardiovascular diseases, particularly for myocardial infarction [1]. It aggravates the susceptibility to myocardial myocardial infarction and abrogates the efficacy of cardioprotective strategies for reducing myocardial injury when ischemia-reperfusion occurs. Indeed, post-conditioning failed to reduce the infarct size in obese mice [2], while conditioning strategies have well-demonstrated their ability to induce a powerful protection against myocardial infarction in the absence of co-morbidities. Among others, the mechanisms involve the activation of pro-survival kinase cascades [3], such as the reperfusion injury salvage kinase (RISK) pathway [3,4,5,6], which includes phosphatidylinositol 3-OH kinase (PI3K) and protein kinase B (Akt). The phosphorylation of PI3K-Akt activates downstream key players, such as protein kinase C (PKC), whose activation is critical for inducing cardioprotection [7,8,9,10,11]. Ischemic preconditioning is associated with selective translocation of the ε isoform of PKC from the cytosol to the membrane fraction, without any changes in total PKC [7]. PKC activation was demonstrated to protect the cardiac mitochondria by inhibiting the opening of the mitochondrial permeability transition pore (mPTP) [12,13], a critical trigger of cell death [14]. In the case of obesity, postconditioning is incapable of increasing Akt phosphorylation to induce cardioprotection [2]. In this context, we previously reported that regular exercise restores cardioprotection in obese mice by increasing the phosphorylation of survival kinases and enhancing the resistance of mPTP to opening at reperfusion [15].

Oxysterols also play a physiopathological role during ischemia-reperfusion, as they can induce apoptosis [16]. They accumulate during early reperfusion of an ischemic myocardium in mice, and regular exercise prevented this accumulation [17]. Among numerous oxysterols, previous studies indicate that 7β-hydroxycholesterol (7βOH) induces apoptosis, and it involves a reduction in Akt activity [18,19]. However, it is not known whether this reduction occurs specifically during the ischemia-reperfusion sequence or it is a pre-emptive mechanism. Therefore, the hypothesis was that regular exercise could increase Akt phosphorylation by reducing 7βOH in the myocardium as a pre-emptive protective mechanism before the occurrence of ischemia-reperfusion.

Using ob/ob mice, we showed, in the present study, that chronic exercise significantly reduced 7βOH myocardial content, and this was accompanied by an increase in Akt phosphorylation, thus showing an activation of the cardioprotective RISK pathway as a pre-emptive mechanism before any ischemic insult. Conversely, cardiomyocytes exposed to 7βOH elicited reduced Akt phosphorylation. Finally, as a translational attempt, we provide results showing a concomitant increase in 7βOH concentration in the serum of obese patients, as compared to healthy volunteers. Concomitantly, Akt phosphorylation in the PBMC issued from their blood was reduced, respectively.

## 2. Results

### 2.1. Effect of Regular Exercise on Signaling Pathways

**Figure 1:**
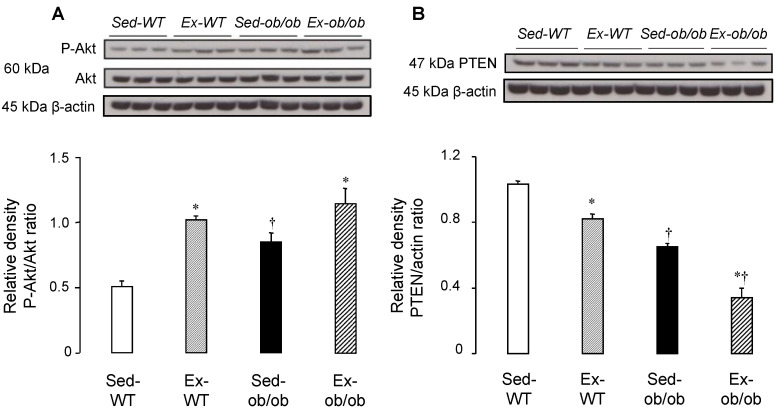
Western blot analysis of Akt and its phosphorylated form (Panel (**A**)), as well as the corresponding phosphatase PTEN (Panel (**B**)), in cytosol in sedentary conditions (Sed) or after regular exercise (Ex) in wild-type (WT) (*n* = 3) or ob/ob (*n* = 3) mice. Values are expressed as mean ± SEM. * *p* < 0.05 vs. corresponding group. † *p* < 0.05 vs. corresponding WT group.

We previously reported that regular exercise was able to induce cardioprotection in both lean and obese mice [15]. We, therefore, investigated the phosphorylation of Akt, a key member of the RISK pathway. Exercise significantly increased the phospho-Akt/Akt ratio in the cytosol of both wild-type (WT) and ob/ob mice (Figure 1A). As some phosphatases are known to inactivate the pro-survival kinases of the RISK pathway, the expression of PTEN, the Akt corresponding phosphatase, was also investigated, and we found a reduced expression of PTEN in both strains, following chronic exercise (Figure 1B).

### 2.2. Translocation of PKCε Following Regular Exercise

**Figure 2:**
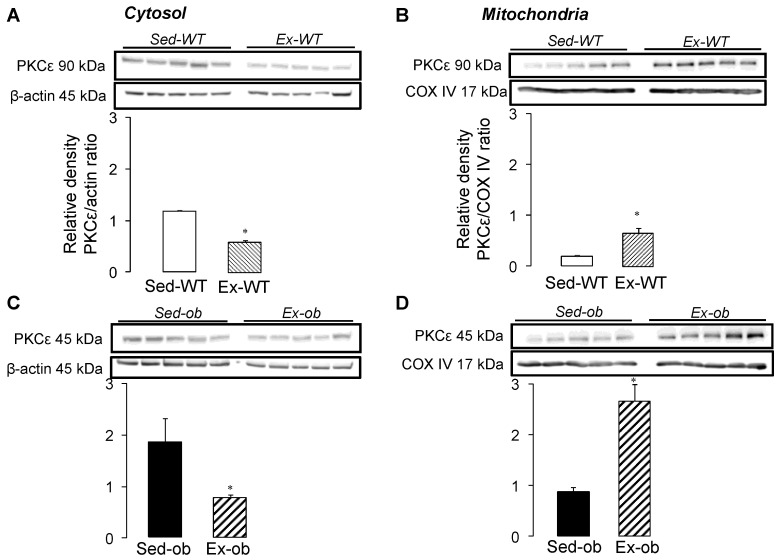
Western blot analysis of PKCε in cytosol (wild-type mice, *n* = 5, Panel (**A**); ob/ob mice, *n* = 5, Panel (**C**)) and in mitochondria (wild-type mice, *n* = 5, Panel (**B**); ob/ob mice, *n* = 5, Panel (**D**)) in sedentary conditions (Sed) or after regular exercise (Ex). Values are expressed as mean ± SEM. * *p* < 0.05 vs. corresponding group.

To investigate the link between the cytosol and mitochondria, we examined the subcellular localization of PKCε, following regular exercise (Figure 2). The expression of PKCε decreased significantly in the cytosol of both the WT (Figure 2A) and ob/ob (Figure 2C) mice, while the levels of PKCε were significantly increased in the mitochondria of the WT (Figure 2B) and obese (Figure 2D) animals. These results suggest an exercise-induced translocation of PKCε from cytosol to mitochondria, PKCε, allowing for the transmission of a protective signal from cytosol to mitochondria in both strains.

### 2.3. Effect of Oxysterols on Signaling Pathways

**Figure 3:**
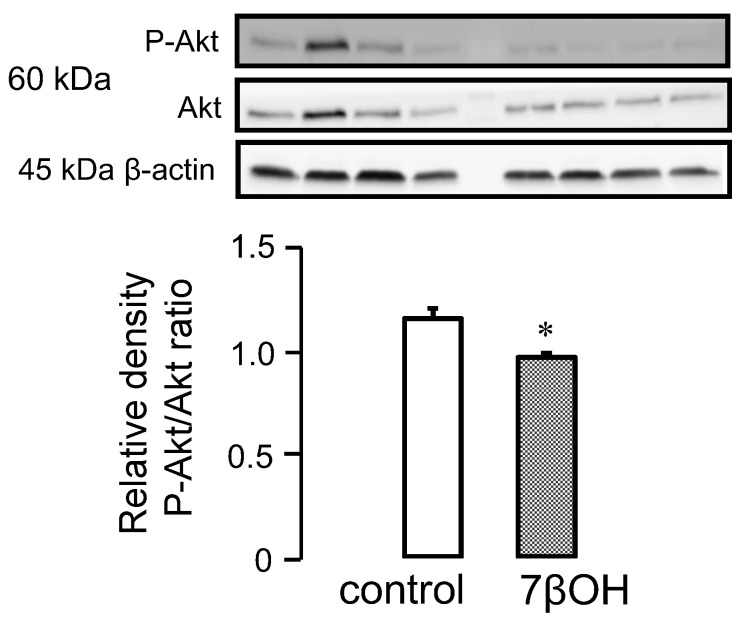
Western blot analysis of Akt and its phosphorylated form in the cytosol of wild-type mice in sedentary conditions (*n* = 4) after incubation of cardiomyocytes with 7β-hydroxycholesterol (7βOH). Values are expressed as mean ± SEM. * *p* < 0.05 vs. corresponding group.

Numerous studies suggest that certain oxysterols could inactivate the pro-survival kinases of the RISK pathway. We, therefore, decided to investigate the effect of 7βOH on cardiomyocytes in WT mice specifically. The phosphorylated Akt/total Akt ratio was significantly reduced in the 7βOH-treated mice cardiomyocytes (Figure 3). This suggests that 7βOH contributes to inactivating the PI3K-Akt signalling pathway.

### 2.4. Effect of Exercise on Oxysterols

**Figure 4:**
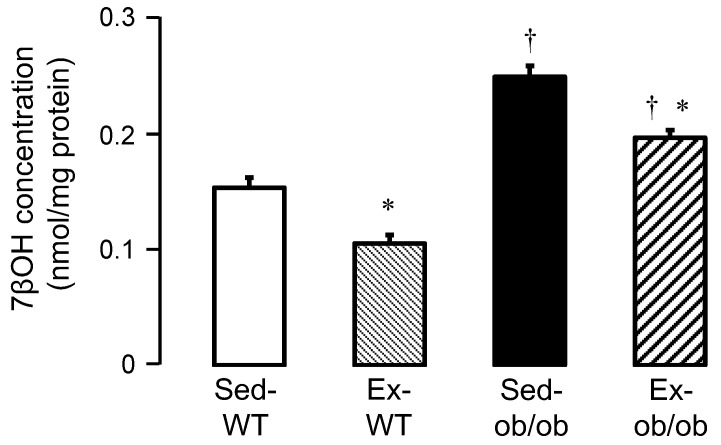
Cytosolic 7β-hydroxycholesterol (7βOH) concentrations in sedentary conditions (Sed) or after regular exercise (Ex) in wild-type (WT, *n* = 9 and 9, respectively) and ob/ob (*n* = 7 and 10, respectively) mice. Values are expressed as mean ± SEM. * *p* < 0.05 vs. corresponding group. † *p* < 0.05 vs. corresponding WT group.

We also investigated the concentrations of 7βOH in the cytosol of WT and ob/ob mice in sedentary and exercise conditions, respectively. The 7βOH cytosolic concentrations significantly increased in sedentary ob/ob mice (Sed-ob/ob), compared to sedentary wild-type mice (Sed-WT mice) (0.2482 ± 0.0095 vs. 0.1529 ± 0.0088 nmol/mg protein, respectively; *p* < 0.05). Exercise significantly reduced the 7βOH cytosolic concentrations in both the Ex-WT and Ex-ob/ob mice (Figure 4).

### 2.5. Effect of Exercice on mPTP Opening

**Figure 5:**
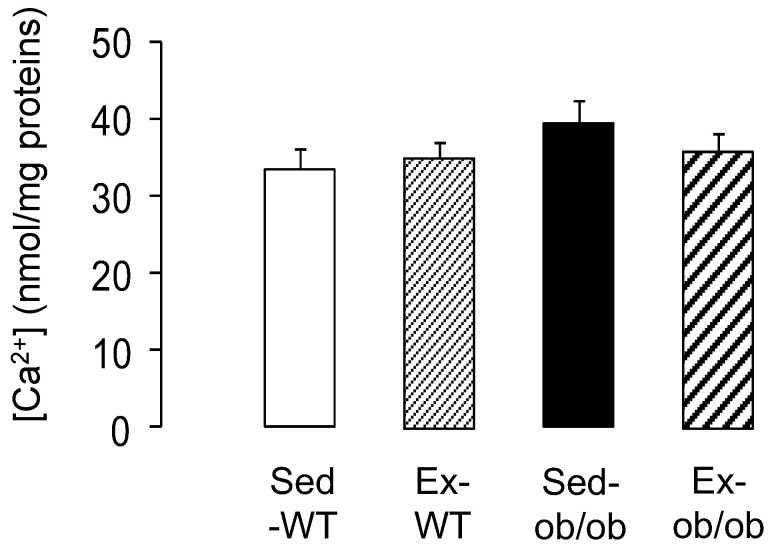
Mitochondrial Ca2+ retention (index of mPTP opening) in wild-type (WT) and ob/ob mice in sedentary conditions (Sed, *n* = 11 and 10, respectively) or after regular exercise (Ex, *n* = 11 and 10, respectively). Values are expressed as mean ± SEM.

As a final target of cardioprotective signaling, we explored mitochondrial susceptibility to calcium overload. Regular exercise did not significantly increase the resistance of the cardiac mitochondria to calcium-induced mPTP opening. Indeed, the calcium concentration required to induce mPTP opening was similar between the WT and ob/ob animals submitted, or not, to the exercise protocol (Figure 5).

### 2.6. Oxysterol Concentrations in Healthy Volunteers and Obese Patients

To investigate whether the oxysterols could be considered candidate as biomarker in patients, nine healthy volunteers (eight females, one male, 41 ± 5 years) and seven obese patients (six females, one male, 38 ± 5 years) were included in the study and experienced oxysterol assays in plasma. Mean body weight was 60 ± 2 kg for healthy volunteers vs. 122 ± 7 kg for obese patients (*p* < 0.05). Mean body mass index was 22.7 ± 0.4 kg/m² for healthy volunteers vs. 42.9 ± 2.2 kg/m² for obese patients (*p* < 0.05). None of the patients had history of cardiac disease.

**Figure 6:**
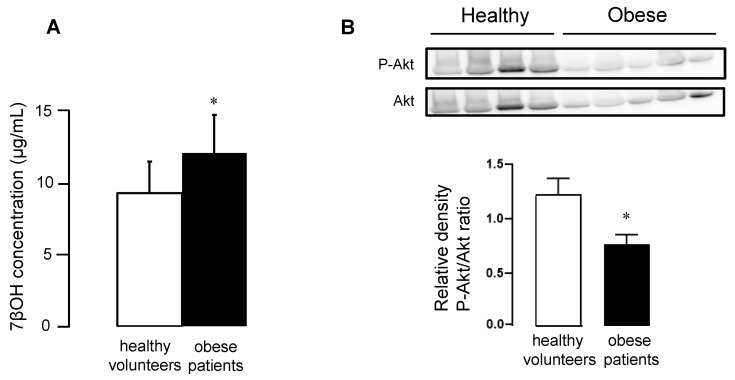
Panel (**A**) Oxysterol concentrations in plasma of sedentary healthy volunteers and obese patients. Panel (**B**) Western blot analysis of Akt and its phosphorylated form in PBMC of sedentary healthy volunteers and obese patients. Values are expressed as mean ± SEM. * *p* < 0.05 vs. corresponding group.

As previously observed in mouse cytosolic samples, concentrations of 7βOH in plasma significantly increased in the obese patients, compared to healthy volunteers (Figure 6A). Moreover, we found a significant decrease in the phosphorylated Akt/total Akt ratios in the peripheral blood mononuclear cells (PBMC) of obese patients, compared to healthy volunteers (Figure 6B).

## 3. Discussion

The present study demonstrates that regular exercise enhanced the phosphorylation of pro-survival kinase Akt, which is involved in the cardioprotective RISK pathway, and induced PKCε translocation from the cytosol to the mitochondria in WT and ob/ob mice. Moreover, exercise reduced the expression of the corresponding phosphatase PTEN, thus reinforcing the phosphorylation tone, and decreased the concentration of the oxysterol 7βOH, contributing to the activation of the RISK pathway. Exercise did not induce pre-emptive adaptation of mitochondrial mPTP opening *per se*. It is, therefore, reasonable to speculate that these phosphorylations and translocations produced a pre-emptive and chronic activation of cardioprotective signaling that will protect mPTP from opening at reperfusion that follows a prolonged ischemic insult. From a translational prospective, we measured the plasma concentrations of 7βOH in obese patients, which were significantly higher, compared to healthy volunteers, in accordance to the observations made in mice, along with reduced Akt phosphorylation in PBMC. This suggests that 7βOH may represent a biomarker candidate that could be investigated in further studies as a biomarker of cardiovascular risk factors in obesity.

To our knowledge, no study has previously investigated the effects of exercise on myocardial pro-survival kinase pathways in the context of obesity *per se*. In the present study, we did not assess myocardial infarction following ischemia-reperfusion, but we previously reported that regular exercise is cardioprotective by reducing the myocardial infarct size in both lean and obese mice using exactly the same experimental protocol [15]. Here, regular exercise improved the phosphorylation state of myocardial Akt, in agreement with the previous studies reporting such activation after exercise in the rat and human skeletal muscle [20,21]. Moreover, Cao et al. [22] highlighted that exercise significantly improved Akt phosphorylation in skeletal muscle in type 2 diabetic rats. The increased phosphorylation of Akt by exercise was accompanied by major effects on phosphatase expression in both strains. Levels of PTEN, which counteracts Akt phosphorylation, were significantly decreased by exercise in WT and ob/ob mice. Thus, regular exercise induced an increase in kinase phosphorylation and concomitant decrease in the corresponding phosphatase in lean and obese animals. This point is of major importance because these phosphatases can limit the efficacy of cardioprotective strategies, as previously reported with pre- and post-conditioning [2,23,24]. The inhibition of these phosphatases was also demonstrated to be highly protective against infarction in the rabbit heart [25,26]. 

Exercise also resulted in a significant decrease in cytosolic 7βOH concentration, which probably contributes to strengthening the activation of the PI3K-Akt signaling pathway. Indeed, the phosphorylated Akt/total Akt ratio was reduced in 7βOH-treated cardiomyocytes, suggesting that 7βOH can inactivate the PI3K-Akt pathway. Although this was not investigated in the present study, one could speculate that the levels of the phosphatase PTEN were increased by 7βOH, thus participating in the decrease in Akt phosphorylation. This requires further investigations to examine these mechanisms. These findings are in agreement with previous reports highlighting the cytotoxicity of oxysterols in other cell types [27,28]. Indeed, the phosphorylated Akt/total Akt ratio was reduced in 7βOH-treated monocytes [18]. Another limitation of this study is that the correlations between the cytosolic levels of 7βOH and both mitochondrial and cardiac function were unfortunately not investigated here. Further studies are needed to deeper determine the myocardial consequences of reducing cytosolic 7βOH concentration with regular exercise in both lean and obese conditions.

The translocation of PKCε from the cytosol to mitochondria, following regular exercise in both WT and ob/ob mice, is also an important result. This phenomenon is probably one of the main triggers for inducing preconditioning [29,30], with PKCε being associated in the mitochondria with components of the mPTP and the inhibition of its opening [31,32]. PKCε could then provide a memory of the protection that persists after the trigger is withdrawn. Kawamura et al. [10] demonstrated that the translocation of PKCε in the membrane fraction persists, even after a 30-min period, following the ischemic preconditioning procedure. This has also been associated with delayed cardioprotection exercise training [33]. However, despite the enhancement of the phosphorylation state of several kinases involved in the RISK pathway, we did not observe any change in mitochondrial resistance to calcium overload in both ob/ob and WT animals following exercise. This indicates that exercise did not induce pre-emptive cardiac mitochondrial functional adaptations, although the activation of the survival pathways was observed. Similarly, Starnes et al. [34] found that exercise did not improve mitochondrial tolerance to calcium overload in rats, while Marcil et al. [35] demonstrated a delayed mPTP opening with exercise training. It is reasonable to speculate that this activation of survival pathways needs to be combined with stress conditions, i.e., ischemia-reperfusion, to initiate the downstream inhibition of the mPTP opening at the onset of reperfusion. One hypothesis could be that the mechanism of exercise-induced cardioprotection involves two phases, i.e., a trigger phase occurring during exercise and characterized by an activation of the pro-survival signaling pathways, and a second phase occurring at the onset of reperfusion and leading to mitochondrial functional adaptations. Indeed, one study suggested that ischemic preconditioning induces two phases of Akt activation [6], the first one occurring immediately after the ischemic preconditioning stimulus, and the second taking place at the onset of myocardial reperfusion. 

Finally, we investigated whether the oxysterols could be proposed as an indirect biomarker that could reflect the phosphorylation state of the protective signaling pathways. We focused on 7βOH, which was identified both in the plasma of subjects included in the study and cytosol of cardiac tissue in mice. Interestingly, plasma concentrations of 7βOH were significantly increased in obese vs. healthy subjects. This was consistent with the observations made in the cardiac cytosol of mice, suggesting that the plasma reflects cytosolic oxysterols of cardiomyocytes. The increase in plasma 7βOH observed in obese patients might explain the decrease in phosphorylated Akt/total Akt ratios found in PBMC. The increased plasma concentration of 7βOH was reported in a population with an increased risk for cardiovascular disease [36]. Another study failed to show increased 7βOH in obese patients [37]. However, the authors reported significant increases in 7βOH serum concentration in female patients with metabolic syndrome, but still not in obese patients. This apparent discrepancy with our study might be due to differences in patient characteristics with higher body mass index ratios in the present study.

In conclusion, regular exercise enhanced the phosphorylation of the pro-survival kinase Akt, induced PKCε mitochondrial translocation, and decreased the corresponding phosphatase levels and 7βOH concentrations in both lean and obese mice, thus contributing to the cardioprotective signaling. Interestingly, 7βOH concentrations in human plasma were consistent with the results observed in the myocardium in mice, suggesting that this oxysterol may constitute a candidate biomarker to evaluate the cardiovascular risk during obesity.

## 4. Materials and Methods

### 4.1. Experimental Protocol in Mice

The experiments were conducted in accordance with the French regulations concerning the care and use of laboratory animals. All experimental procedures were approved by the ANSES/ENVA/UPEC (Agence Nationale de Sécurité Sanitaire/Ecole Nationale Vétérinaire d’Alfort/Université Paris-Est Créteil) Animal Ethics Committee (approval number COMETH#16—11/12/12-7). Male 8- to 10-week-old wild-type C57BL/6J (WT) and obese (ob/ob) mice were used (R. Janvier, Le Genest Saint Isle, France). Mice were housed in an air-conditioned room with a 12-h light-dark cycle and received standard rodent chow and drinking water ad libitum. 

Both WT (*n* = 55) and ob/ob (*n* = 52) mice were randomly subjected to treadmill exercise (Ex-WT and Ex-ob/ob) or sedentary conditions (Sed-WT and Sed-ob/ob). Exercised animals ran 5 consecutive days per week for 4 weeks. The first week was a 30-min adaptation period with gradual speeds (10, 14, 18, 22, 26, and 30 cm/s, 4° slope, 5 min each step for WT and 8, 8, 10, 14, 18, and 20 cm/s, 4° slope, 5 min each step for ob/ob, 4° slope). For the three other weeks, mice ran during one hour per day (30 and 20 cm/s for WT and ob/ob, respectively, 4° slope). To take into account the differences in body weight [18], the speed was different between the two strains, in order to obtain similar vertical work [38]. The training protocol was well-tolerated by the WT and ob/ob animals and both strains were able to complete the exercise protocol. After these 4 weeks of exercise or sedentary conditions, western blot experiments and oxysterol assays were performed, and mitochondrial function was investigated. We previously showed the infarct limiting properties of regular exercise in both WT and ob/ob mice [15].

### 4.2. Healthy Volunteers and Obese Patients 

Healthy volunteers and obese patients were included after approval of an institutional review board (CPP-IDF IX, approval number 12-003, 2012) and competent health authorities. All subjects included provided written informed consent before participating in the study. Blood samples were collected on lithium heparinate and then rapidly centrifuged, and the plasma was frozen at −80 °C until the oxysterol assays. Peripheral blood mononuclear cells (PBMC) were freshly isolated from whole blood collected on lithium heparinate by Ficoll density gradient. Cell recovery and viability were assessed using a hemacytometer and trypan blue.

### 4.3. Isolation of Adult Mouse Cardiomyocytes

Adult mice were anesthetized with an intraperitoneal injection of sodium pentobarbital (90 mg/kg) and heparin (500 IU). The heart was quickly excised, placed in an ice-cold calcium-free perfusion buffer, and first perfused using a Langendorff apparatus with the perfusion buffer at 37 °C. Then, a digestion buffer containing calcium, liberase, and trypsin was used. Once enzyme digestion of the heart was completed, the left ventricle was dissected. It was then minced with scissors and homogenised with a pipette in a stopping buffer containing calcium and 10% bovine calf serum. The cellular pellet was washed 6 times with a perfusion buffer containing 5% bovine calf serum and increasing concentrations of calcium. If the final viability was greater than 65%, these adult mouse cardiomyocytes were then incubated with 25 µM of 7β-hydroxycholesterol (7βOH) solubilized in ethanol during 24 h at 37 °C and 5% CO_2_ in a humidified incubator. This concentration of 7βOH has been chosen on the basis of preliminary experiments guided by previous reports [18,19].

### 4.4. Cytosol Preparation from Isolated Adult Mice Cardiomyocytes and Human PBMC

Adult mouse cardiomyocytes and human PBMC, when available, were collected by centrifugation at 1000× *g* for 10 min at 4 °C. Cell pellet was resuspended in a homogenization buffer containing 220 mM mannitol, 70 mM sucrose, 10 mM HEPES, 1 mM EGTA and supplemented with protease and phosphatase inhibitors (pH 7.4 at 4 °C). Two cycles freezing-thawing at −80 °C, followed by 5 sonication cycles, were then used to break the cell membranes. A final centrifugation at 16,100× *g* for 1 h at 4 °C allowed for collecting the cytosol.

### 4.5. Preparation of Cytosols and Mitochondria from Fresh Ventricular Tissues

Cytosol and mitochondria were extracted from fresh left ventricular tissue. The tissues were scissor minced, homogenized on ice using a Teflon Potter homogenizer, and centrifuged at 1000× *g* for 5 min. Supernatants were centrifuged at 10,000× *g* for 10 min at 4 °C. The final mitochondrial pellet was then resuspended in 150 µL of a homogenization buffer containing 220 mM mannitol, 70 mM sucrose, 10 mM HEPES, 1 mM EGTA, and supplemented with protease and phosphatase inhibitors (pH 7.4 at 4 °C). The supernatants were centrifuged at 100,000× *g* for 60 min at 4 °C. The mitochondrial and cytosolic protein concentrations were determined by the BCA protein assay kit (Pierce, Rockford, IL, USA).

### 4.6. Evaluation of mPTP Opening

To measure mitochondrial calcium retention as an index of the resistance to calcium-induced mPTP opening, cardiac mitochondria (0.8 mg/mL), incubated with 3.3 µM calcium Green-5N fluorescent probe (Interchim, Montluçon, France) were loaded by the addition of successive 2.5 µM calcium pulses. When maximal calcium-loading threshold was reached, mitochondria underwent a fast process of calcium release due to mPTP opening. The concentration of calcium in the extra-mitochondrial medium was monitored by means of a Perkin Elmer LS 50B fluorescence spectrometer at excitation and emission wavelengths of 506 and 532 nm, respectively.

### 4.7. Western Blot Analysis

Western blot analyses were performed to investigate phosphorylated Akt, total Akt, PTEN, and PKCε. To extract proteins, myocardial tissues were placed in medium containing 220 mM mannitol, 70 mM sucrose, 10 mM HEPES, 1 mM EGTA, 5µL/mL of protease inhibitor cocktail, 1 mM sodium orthovanadate, 5 mM sodium fluoride, 1 mM sodium Na2β-glycerol phosphate, and pH 7.4 at 4 °C. Extracted protein samples were denatured at 95 °C for 5 min. Proteins were separated by SDS-polyacrylamide gel electrophoresis, transferred to nitrocellulose membranes, and probed with primary antibodies for β-actin (1/2000, Sigma Aldrich, Saint Quentin Fallavier, France), phospho-Akt (Serine 473, 1/1000), total Akt (1/2000), PTEN (1/1000) (Cell Signaling Technology, Danvers, MA, USA), and PKCε (1/1000, Santa Cruz Biotechnology, Dallas, TX, USA). Blots were washed and then incubated with anti-rabbit IgG secondary antibody (1/2000, Santa Cruz Biotechnology, Dallas, TX, USA). Bands of interest were scanned and quantified in a blinded manner using gel analysis software ImageJ-1.37 (National Institute of Health, Bethesda, ML, USA).

### 4.8. Oxysterol Assays

Oxysterol measurements were performed on cytosol extracts from mouse fresh cardiac tissue and plasma samples from human subjects. Concentrations of 7β-hydroxycholesterol (7βOH) were measured by gas chromatography-mass spectrometry (GC-MS) [39,40]. D7-7β-hydroxycholesterol was used as an internal standard. Cytosols were treated with 50 µL of butylated hydroxytoluene (5 g/L) and 50 µL of EDTA (10 g/L) to prevent auto oxidation. Each vial was then flushed with argon for 20 min to remove air. Alkaline hydrolysis was allowed to proceed at room temperature (22 °C), with magnetic stirring for 1 h in the presence of ethanolic 1 M potassium hydroxide solution. After hydrolysis, the sterols were extracted twice with 5 mL cyclohexane and oxysterols were eluted on SPE cartridge by isopropanol/hexane 30/70 *v*/*v*. The organic solvents were evaporated under a gentle stream of argon and converted into trimethylsilyl ethers [41].

### 4.9. Statistical Analysis

Statistical analysis was performed using Kruskal–Wallis for overall analysis followed by individual comparisons using Mann–Whitney tests, with significance at *p* < 0.05. Non-parametric tests were used, with respect of the low number of animals in each experiment.

## Data Availability

Any data or material that support the findings of this study can be made available by the corresponding author upon request.

## References

[1] Heusch G. (2006). Obesity—A risk factor or a RISK factor for myocardial infarction?. Br. J. Pharmacol..

[2] Bouhidel O., Pons S., Souktani R., Zini R., Berdeaux A., Ghaleh B. (2008). Myocardial ischemic postconditioning against ischemia-reperfusion is impaired in ob/ob mice. Am. J. Physiol. Heart Circ. Physiol..

[3] Hausenloy D. (2004). New directions for protecting the heart against ischaemia–reperfusion injury: Targeting the Reperfusion Injury Salvage Kinase (RISK)-pathway. Cardiovasc. Res..

[4] Hausenloy D. (2004). Cross-talk between the survival kinases during early reperfusion: Its contribution to ischemic preconditioning. Cardiovasc. Res..

[5] Hausenloy D.J., Yellon D.M. (2007). Reperfusion injury salvage kinase signalling: Taking a RISK for cardioprotection. Heart Fail. Rev..

[6] Hausenloy D.J., Tsang A., Mocanu M.M., Yellon D.M. (2005). Ischemic preconditioning protects by activating prosurvival kinases at reperfusion. Am. J. Physiol. Heart Circ. Physiol..

[7] Ping P., Zhang J., Qiu Y., Tang X.L., Manchikalapudi S., Cao X., Bolli R. (1997). Ischemic preconditioning induces selective translocation of protein kinase C isoforms ε and η in the heart of conscious rabbits without subcellular redistribution of total protein kinase C activity. Circ. Res..

[8] Ytrehus K., Liu Y., Downey J.M. (1994). Preconditioning protects ischemic rabbit heart by protein kinase C activation. Am. J. Physol. Heart Circ. Physiol..

[9] Liu Y., Ytrehus K., Downey J.M. (1994). Evidence that translocation of protein kinase C is a key event during ischemic preconditioning of rabbit myocardium. J. Mol. Cell. Cardiol..

[10] Kawamura S., Yoshida K.I., Miura T., Mizukami Y., Matsuzaki M. (1998). Ischemic preconditioning translocates PKC-δ and -ε, which mediate functional protection in isolated rat heart. Am. J. Physiol. Heart Circ. Physiol..

[11] Tong H., Chen W., Steenbergen C., Murphy E. (2000). Ischemic preconditioning activates phosphatidylinositol-3-kinase upstream of protein kinase C. Circ. Res..

[12] Hausenloy D., Yellon D. (2006). Survival kinases in ischemic preconditioning and postconditioning. Cardiovasc. Res..

[13] Downey J.M., Davis A.M., Cohen M.V. (2007). Signaling pathways in ischemic preconditioning. Heart Fail Rev..

[14] Hausenloy D., Yellon D. (2003). The mitochondrial permeability transition pore: Its fundamental role in mediating cell death during ischaemia and reperfusion. J. Mol. Cell. Cardiol..

[15] Pons S., Martin V., Portal L., Zini R., Morin D., Berdeaux A., Ghaleh B. (2013). Regular treadmill exercise restores cardioprotective signaling pathways in obese mice independently from improvement in associated co-morbidities. J. Mol. Cell. Cardiol..

[16] Lordan S., Mackrill J.J., O’Brien N.M. (2009). Oxysterols and mechanisms of apoptotic signaling: Implications in the pathology of degenerative diseases. J. Nutr. Biochem..

[17] Musman J., Pons S., Barau C., Caccia C., Leoni V., Berdeaux A., Ghaleh B., Morin D. (2016). Regular treadmill exercise inhibits mitochondrial accumulation of cholesterol and oxysterols during myocardial ischemia-reperfusion in wild-type and ob/ob mice. Free Radic. Biol. Med..

[18] Lordan S., O’Neill C., O’Brien N.M. (2008). Effects of apigenin, lycopene and astaxanthin on 7β-hydroxycholesterol-induced apoptosis and Akt phosphorylation in U937 cells. Br. J. Nutr..

[19] Clarion L., Schindler M., de Weille J., Lolmède K., Laroche-Clary A., Uro-Coste E., Robert J., Mersel M., Bakalara N. (2012). 7β-Hydroxycholesterol-induced energy stress leads to sequential opposing signaling responses and to death of c6 glioblastoma cells. Biochem. Pharmacol..

[20] Boonsong T., Norton L., Chokkalingam K., Jewell K., Macdonald I., Bennett A., Tsintzas K. (2007). Effect of exercise and insulin on SREBP-1c expression in human skeletal muscle: Potential roles for the ERK1/2 and Akt signalling pathways. Biochem. Soc. Trans..

[21] Sakamoto K., Aschenbach W.G., Hirshman M.F., Goodyear L.J. (2003). Akt signaling in skeletal muscle: Regulation by exercise and passive stretch. Am. J. Physiol. Endocrinol. Metab..

[22] Cao S., Li B., Yi X., Chang B., Zhu B., Lian Z., Zhang Z., Zhao G., Liu H., Zhang H. (2012). Effects of exercise on AMPK signaling and downstream components to PI3K in rat with type 2 diabetes. PLoS ONE.

[23] Fenton R.A., Dickson E.W., Dobson J.G. (2005). Inhibition of phosphatase activity enhances preconditioning and limits cell death in the ischemic/reperfused aged rat heart. Life Sci..

[24] Przyklenk K., Maynard M., Darling C.E., Whittaker P. (2008). Aging mouse hearts are refractory to infarct size reduction with post-conditioning. J. Am. Coll. Cardiol..

[25] Weinbrenner C., Baines C.P., Liu G.S., Armstrong S.C., Ganote C.E., Walsh A.H., Honkanen R.E., Cohen M.V., Downey J.M. (1998). Fostriecin, an inhibitor of protein phosphatase 2A, limits myocardial infarct size even when administered after onset of ischemia. Circulation.

[26] Armstrong S., Gao W., Lane J., Ganote C. (1998). Protein phosphatase inhibitors calyculin A and fostriecin protect rabbit cardiomyocytes in late ischemia. J. Mol. Cell. Cardiol..

[27] Sghaier R., Zarrouk A., Nury T., Badreddine I., O’Brien N., Mackrill J.J., Vejux A., Samadi M., Nasser B., Caccia C. (2019). Biotin attenuation of oxidative stress, mitochondrial dysfunction, lipid metabolism alteration and 7β-hydroxycholesterol-induced cell death in 158N murine oligodendrocytes. Free Radic. Res..

[28] Ghzaiel I., Zarrouk A., Essadek S., Martine L., Hammouda S., Yammine A., Ksila M., Nury T., Meddeb W., Tahri Joutey M. (2022). Protective effects of milk thistle (*Sylibum marianum*) seed oil and α-tocopherol against 7β-hydroxycholesterol-induced peroxisomal alterations in murine C2C12 myoblasts: Nutritional insights associated with the concept of pexotherapy. Steroids.

[29] Melling C.W.J., Thorp D.B., Milne K.J., Noble E.G. (2009). Myocardial Hsp70 phosphorylation and PKC-mediated cardioprotection following exercise. Cell Stress Chaperones.

[30] Carson L.D., Korzick D.H. (2003). Dose-dependent effects of acute exercise on PKC levels in rat heart: Is PKC the heart’s prophylactic? Signal transduction and exercise-induced cardioprotection. Acta Physiol. Scand..

[31] Korge P., Honda H.M., Weiss J.N. (2002). Protection of cardiac mitochondria by diazoxide and protein kinase C: Implications for ischemic preconditioning. Proc. Natl. Acad. Sci. USA.

[32] Baines C.P., Song C.X., Zheng Y.T., Wang G.W., Zhang J., Wang O.L., Guo Y., Bolli R., Cardwell E.M., Ping P. (2003). Protein Kinase Cε interacts with and inhibits the permeability transition pore in cardiac mitochondria. Circ. Res..

[33] Ghosh S., Khazaei M., Moien-Afshari F., Ang L.S., Granville D.J., Verchere C.B., Dunn S.R., Mc Cue P., Mizisin A., Sharma K. (2009). Moderate exercise attenuates caspase-3 activity, oxidative stress, and inhibits progression of diabetic renal disease in db/db mice. Am. J. Physiol. Ren. Physiol..

[34] Starnes J.W., Barnes B.D., Olsen M.E. (2007). Exercise training decreases rat heart mitochondria free radical generation but does not prevent Ca^2+^-induced dysfunction. J. Appl. Physiol..

[35] Marcil M., Bourduas K., Ascah A., Burelle Y. (2006). Exercise training induces respiratory substrate-specific decrease in Ca^2+^-induced permeability transition pore opening in heart mitochondria. Am. J. Physiol. Heart Circ Physiol..

[36] Ziedén B., Kaminskas A., Kristenson M., Kucinskienê Z., Vessby B., Olsson A.G., Diczfalusy U. (1999). Increased plasma 7β-hydroxycholesterol concentrations in a population with a high risk for cardiovascular disease. Arterioscler. Thromb. Vasc. Biol..

[37] Tremblay-Franco M., Zerbinati C., Pacelli A., Palmaccio G., Lubrano C., Ducheix S., Guillou H., Iuliano L. (2015). Effect of obesity and metabolic syndrome on plasma oxysterols and fatty acids in human. Steroids.

[38] Massett M.P., Berk B.C. (2005). Strain-dependent differences in responses to exercise training in inbred and hybrid mice. Am. J. Physiol. Regul. Integr. Comp. Physiol..

[39] Leoni V., Caccia C. (2011). Oxysterols as biomarkers in neurodegenerative diseases. Chem. Phys. Lipids.

[40] Dzeletovic S., Breuer O., Lund E., Diczfalusy U. (1995). Determination of cholesterol oxidation products in human plasma by isotope dilution-mass spectrometry. Anal. Biochem..

[41] Paradis S., Leoni V., Caccia C., Berdeaux A., Morin D. (2013). Cardioprotection by the TSPO ligand 4′-chlorodiazepam is associated with inhibition of mitochondrial accumulation of cholesterol at reperfusion. Cardiovasc. Res..

